# Relationship between Calcium Score and Myocardial Scintigraphy in the Diagnosis of Coronary Disease

**DOI:** 10.5935/abc.20160104

**Published:** 2016-10

**Authors:** Fabio Paiva Rossini Siqueira, Claudio Tinoco Mesquita, Alair Augusto Sarmet M. Damas dos Santos, Marcelo Souto Nacif

**Affiliations:** 1 Programa de Pós-graduação em Ciências Cardiovasculares da Universidade Federal Fluminense (UFF) Niterói, RJ - Brazil; 2 Setor de Medicina Nuclear, Hospital Universitário Antônio Pedro (HUAP), Universidade Federal Fluminense (UFF) Niterói, RJ - Brazil; 3 Departamento de Radiologia, Faculdade de Medicina, Universidade Federal Fluminense (UFF); 4 Serviço de Radiologia, Hospital Universitário Antônio Pedro (HUAP), Universidade Federal Fluminense (UFF) Niterói, RJ - Brazil

**Keywords:** Coronary Artery Disease / diagnoses, Scintigraphy, Calcium Signaling, Tomography, Emission Computed

## Abstract

Half the patients with coronary artery disease present with sudden death - or acute infarction as first symptom, making early diagnosis pivotal. Myocardial perfusion scintigraphy is frequently used in the assessment of these patients, but it does not detect the disease without flow restriction, exposes the patient to high levels of radiation and is costly. On the other hand, with less radiological exposure, calcium score is directly correlated to the presence and extension of coronary atherosclerosis, and also to the risk of cardiovascular events. Even though calcium score is a tried-and-true method for stratification of asymptomatic patients, its use is still reduced in this context, since current guidelines are contradictory to its use on symptomatic diseases. The aim of this review is to identify, on patients under investigation for coronary artery disease, the main evidence of the use of calcium score associated with functional evaluation and scintigraphy.

## Introduction

Cardiovascular diseases (CVDs) remain the primary cause of death in Brazil and in the world, accounting for over 30% of total deaths on the planet - of which 50% are related to coronary artery disease (CAD). According to the World Health Organization, in 2008, approximately 17 million deaths were related to cardiovascular system disorders and, though some regions showed a drop in these rates, absolute numbers are still alarming.^1^

According to the American Heart Association, in 2009, one in every six hospital admissions in the United States was due to cardiovascular diseases, amounting to 6 million patients. It is estimated that, only in USA, 15 million people suffer from coronary disorders, and data from 2004 show that admissions and procedures related to coronary artery disease account for over 44 billion dollars.^2^

Over the last decades, it has been observed that the progression of CVDs was greater in developing countries in comparison to developed ones.^1^ A large portion of deaths happens in underdeveloped regions, being four to five times more frequent. This shows that, in Latin America, the epidemiologic transition of cardiovascular diseases is in a different stage than in North America or Western Europe.^3^

In Brazil, statistics point in the same direction. CVDs are still the main cause of death, in men and women, accounting for around 20% of all deaths in the country. According to the Brazilian Ministry of Health, in 2009, over 139,000 deaths were caused by disorders related to atherosclerosis.^4^ Incidence of cerebrovascular disease is still superior to that of coronary disease, which suggests that CAD, over the next few decades, may become more frequent in our population and be the main cause of death, if the epidemiologic transition follows the same path as in developed nations.^5^

### Pathophysiology of atherosclerosis

The main cause of coronary insufficiency is atherosclerotic disease, defined as an inflammatory disorder. Plaque formation begins with early accumulation of low density lipoprotein particles (LDL) in the arterial intima.^6^ Oxidation of lipid material is one of the factors responsible for the attack on the endothelium, altering its permeability and increasing the expression of adhesion molecules, integrins and selectins, which participate in the migration of monocytes as part of the innate inflammatory response.^7^

Macrophages initiate LDL phagocytosis, which results in the formation of foam cells that produce cytokines and metalloproteinases, amplifies inflammatory response and recruits platelets and T lymphocytes.^6,7^ Platelets adhere to the lesion and release prostaglandins and leukotrienes, as well as growth factors that induce monocytes and smooth muscle cell multiplication.^7^ T lymphocytes are presented to lesion antigens by dendritic cells, start producing cytokines, and modulate adaptive immune response.^6^

Deposition of extracellular matrix produced by the differentiated smooth muscle, cell proliferation, necrosis, and angiogenesis promote expansion of the plaque.^8^ The progression of atherosclerotic disease, however, does not obstruct vascular light in the same proportion due to positive remodeling of vessel size which does not compromise the luminal diameter.^8,9^ When maximum capacity is reached, we see negative remodeling and plaque progression to the interior of the artery which, by gradually compromising the flow, may cause myocardial ischemia.^8^

Migration and proliferation of poorly differentiated smooth muscle cells in the intima promotes atherosclerotic plaque mineralization.^9^ These cells are able to differentiate into osteoblasts, produce mineralized extracellular matrix, and deposit hydroxyapatite crystals by accumulating calcium in the interior of the lesion^9^,^10^ in an osteogenesis-like process. Microcalcifications and calcified deposits may lead to plaque cavitation, erosion and rupture, increasing the risk of coronary thrombosis.^10^

### Coronary artery disease assessment

Despite the slow progression of atherosclerotic disease in the intima of the coronary arteries (it takes years for precursor lesions to become a plaque that causes luminal obstruction), the possibility of becoming unstable and rupturing the plaque makes atherosclerosis, even in its subclinical form, a risk factor for the occurrence of acute coronary events.^11^

Approximately 50% of patients with CAD present with sudden death or acute myocardial infarction (AMI), making early diagnosis a pivotal factor. Early detection through joint evaluation of clinical and lab data and risk factors, associated to non-invasive imaging tests in selected patients, allows the applications of the best prevention and risk stratification strategies.^12^

Multiple risk factors have traditionally been associated to the occurrence of acute events in patients with CAD, and may be clinically evaluated through cardiovascular risk scores. However, this exclusively clinical approach, has proved limited in the diagnosis and prediction of events such as AMI and sudden death, when compared to risk prediction through associated supplementary methods.^13^

In the last few decades, several diagnostic methods to estimate cardiovascular risk and diagnose subclinical atherosclerosis in asymptomatic patients have been studied. Among them, coronary calcium score (CS) has shown excellent accuracy in the prediction of future risk events and detection of early disease that may be isolated or associated to clinical scores.^14^

Calcium score capacity for cardiovascular risk stratification has also been compared to the so-called new risk factor, capable of estimating subclinical atherosclerosis, such as carotid intimal-medial layer thickening measurement, ankle-brachial index, and C-reactive protein. However, coronary calcification was more effective in the process of re-rating patient groups' risk by clinical score, even when used alone.^15-17^

On the other hand, non-invasive stratification of symptomatic patients relies on more advanced methods for anatomic and functional studies. Even though there are several proven accurate methods of coronary disease detection, myocardial perfusion scintigraphy (MPS) is the most frequently requested for diagnostic and prognostic evaluation of these patients in clinical practice.^18^

### Myocardial perfusion scintigraphy

Myocardial perfusion scintigraphy was introduced in the 70's for cardiac perfusion and ventricular function assessment.^19^ Through the years, due to vast literature of accuracy evidence, its diagnostic and prognostic value and its cost-effectiveness, this technique has evolved into an important risk stratification tool and cardiovascular event predictor, and become one of the most utilized non-invasive methods in cardiology.^19-21^

Scintigraphy is a method based on image formation through the acquisition of photons emitted by radiopharmaceuticals and captured by detectors located in a structure called gamma-chamber, with technetium-99m and thallium chloride -201^22,23^ as the main radioactive substances (radioisotopes) used to provide images of the heart. Myocardial perfusion data are reconstructed into multiplanar images, through specific software, which correspond to cardiac tissue perfusion at the moment of medication administration.^24^

The exam is performed in two distinct moments: at rest and during effort - in one or two-day protocols. The effort may be physical or pharmacological through the use of vasodilating substances or inotropic agents. Such exam is able to assess myocardial perfusion by comparing the acquired images at rest and during effort and detect reversible perfusion defects, suggestive of ischemia. Moreover, it provides information on myocardial viability, cardiac function, ejection fraction, cavity volumes, and ventricular synchronicity.^23-25^

Over the last few decades, a lot of effort has been put into the technique development for optimization and patient safety improvement. Chambers with cadmium zinc telluride (CZT) detectors have shown improvements in image quality, less exposure to radiation, and reduced time for acquisition, showing great progress in relation to traditional chambers. Other than gamma-chamber advancements, electronic image-treatment programs, iterative reconstruction in particular, also yield better quality exams using lower doses of radiotracers.^26,27^

Myocardial perfusion scintigraphy is an established and consolidated technique in ischemia detection and prognostic evaluation. The exam shows great ability in the detection of lesions with flow restriction.^28,29^ The same method is also able to determine patient prognosis - a regular exam is usually associated with less than 1% of adverse cardiovascular events per year, whereas exams with evidence of ischemia indicate increased risk, proportionate to defect extension.^30^

### Coronary calcium score

Calcium presence in coronary arteries is a strong indicator of CAD. It has thus arisen great interest in its possibilities of diagnostic and prognostic application since it was described and used as a coronary disease detection technique, initially through fluoroscopy and electron beam CT in the 70's.^31-33^

Since the introduction of computerized multiple detector CT in the 80's, it has been adapted for these new machines, yielding similar results and showing superior results in some cases, when systems with 64 detectors or more are available.^32-34^

Calcium score is obtained from the acquisition of axial chest images, synchronized to the electrocardiogram, with 3 mm thick slices, without the use of iodinated contrast. In general, the effective dose of radiation is reduced and may vary with the characteristics of the scanner and technique used.^32-35^

Hyperattenuating lesions with signal strength above 130 Hounsfield units and area equal to or greater than 3 contiguous pixels are quantified. Total volume, mass and Agatston score-weighted sum may be provided. The technique described by Agatston et al. is the most used in literature and the one presenting the most evidence.^32-35^

Patients may be divided into groups according to the extension of the disease: Absence of calcification; minimal calcification (1-10); mild (11-100); moderate (101-400); severe (401-1000); and extensive (more than 1000). Patients may also be divided according to percentile of age, gender, and ethnicity.^35^

Initially, a series of studies addressed the calcium score's ability to predict the presence of significant anatomical lesions in invasive coronary angiography and its role in acute coronary syndromes as triage for catheterization.^31,32,36^ Throughout the years, the focus switched to prognostic power and cardiovascular event occurrence prediction ability.^33,37-40^

Several studies have shown that, as an indicator of atherosclerosis and cardiovascular risk, that is independent and superior to other methods and clinical scores,^33,37-40^ the presence of coronary calcification is correlated to cardiovascular events.

Risk of patients with CS above 1000 can be 12 times greater, and even minimal calcification presents an increased risk of 2 to 3 fold.^39^

On the other hand, in the presence of a score of zero, there is a small probability of disease in patients with low to intermediate risk, even in those showing symptoms.^37,38^ Moreover, it denotes that the disease does not show great extent, which is a good prognostic indicator. The absence of calcium in the coronary determines an annual risk close to 0.1%.^40^

MPS is an excellent method to assess obstructive disease and indicates a good prognosis when negative; however, it fails to identify lesions without flow restriction.^27^ Moreover, it is costly, uses larger doses of ionizing radiation than calcium scores, requires specific technological apparatus, specialized staff, and supply of radiological material.^24^

Conversely, SC is directly related to the presence and extension of coronary atherosclerosis. Furthermore, it is potentially more widely available and has a lower cost in relation to scintigraphy, as well as a smaller effective radiation dose. However, it is not capable of identifying coronary stenosis and its role remains undefined in the symptomatic disease.^37^

Information provided by the methods are possibly complementary, which allows a joint approach. However, the correlation between the results, the influence of population characteristics on the findings and the sequential use of methods are not definitely established.^38-40^ Therefore, so far there is no consensus of clinical guidelines on the joint application of calcium score and scintigraphy.

### Correlation between methods

A few decades ago, a special interest arose on the accuracy of non-invasive methods for the detection of coronary artery atherosclerosis, with an attempt to establish a parallel between the numerous forms of coronary approach. The ability of CS to identify patients at risk of ischemia using myocardial scintigraphy has been addressed by several studies in literature.^41,50^

Results suggest a correlation between total coronary calcium and scintigraphy, showing that higher calcium scores mean more frequent perfusion defects and more severe ischemia in the area.^41,42^ The opposite applies, with a lessened incidence of ischemia in patients with a lower calcium score.^41-43^

In general, the increase of coronary calcification is correlated to a greater occurrence of ischemia. Patients with metabolic disorders and presence of coronary calcium are also more likely to have perfusion abnormalities than patients without comorbidities.^44^ In the diabetic population, the calcium score shows correlation to the presence of alterations in scintigraphy in a manner superior to traditional risk factors.^45^

Despite the described evidence, the correlation between calcium scores and myocardial perfusion may vary according to populational characteristics and symptoms.^47^ Clinical presentation shows great significance in the correlation between methods. In patients at high risk for CAD, occurrence of alterations in the functional exam, even with reduced calcification, was more frequent in relation to those at low or intermediate risk.^47,48^

A coronary layer analysis shows that, in low calcium score coronaries, the presence of ischemia in its territories was significantly lower, with similar predictive values to CT angiography.^49,50^ Even though calcium score is related to the extension of the disease and not to the stenosis level, it showed similar ability to CT angiography in the prediction of myocardial perfusion alterations.^49^

### Calcium score zero

Calcium score, besides adding supplementary information to scintigraphy, is a powerful tool in the assessment of coronary disease. However, some situations stand out, as calcium score zero.^36-40^ Absence of calcium in the coronary arteries does not mean absence of atherosclerosis, as there may be non-calcified plaques. However, this situation correlates to a disease of lower extension.^39,40^

Although the use of CS in asymptomatic patients is included in more recent guidelines, that is not the case for symptomatic patients.^16-18^ However, in exams like scintigraphy,^51^ literature indicates that, in low or intermediate symptoms and risk of coronary disease, a score of zero is able to deviate the presence of perfusion alterations.

As from initial studies, there has been particular interest in calcium score ability to deviate CAD and correlate with normal myocardial perfusion. In a normal functional examination, the absence of coronary calcification is a strong indication of the absence of atherosclerotic disease. When compared to coronary angiography, the presence of significant lesions, the need for intervention, or the occurrence of AMI are unlikely.^47,51-54^

**Figure 1:**
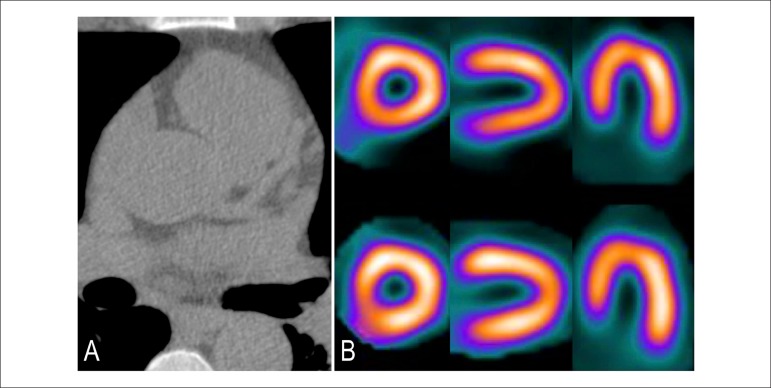
Patient with calcium score zero. (A) Absence of calcified plaques. Risk of coronary disease below 5% and low risk of cardiovascular events (0,1% per year). (B) Normal myocardial scintigraphy.

The absence of coronary calcium has been shown to ward off ischemia caused by CAD in patients with low or intermediate symptoms and probability of significant diseases, and CS, when used in conjunction with scintigraphy, shows increase in specificity and positive predictive value of the diagnostic strategy (Figure 1).^51,55,56^

However, in the acute presentation of symptoms in the emergency room or in high-risk patients for CAD, the use of CS is limited, since atherothrombotic phenomena of acute coronary syndromes may be present without calcification. Absence of calcium in symptomatic patients with coronary angiography indication does not exclude the presence of significant lesions.^57^ In such cases, CS does not add diagnostic and prognostic information, and has lower event prediction power than scintigraphy.^58-60^

### Elevated coronary calcification

Severe coronary calcification has proven to be an independent risk predictor and it is complementary to virtually all other forms of coronary artery disease evaluation, be it clinical - through risk scores - or complementary - via other non-invasive methods and functional tests - as exercise stress test and scintigraphy.^61-68^

**Figure 2:**
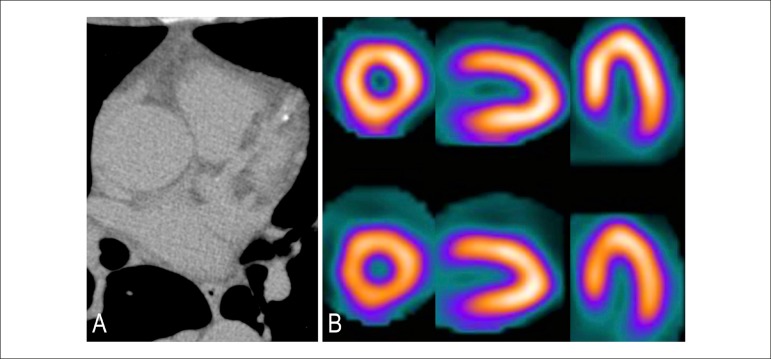
Patient with calcium score 1-10. (A) Minimal quantity of calcified plaques in the territory of the anterior descending artery. Probable risk (obstructive coronary disease below 10%). (B) Normal myocardial scintigraphy.

**Figure 3:**
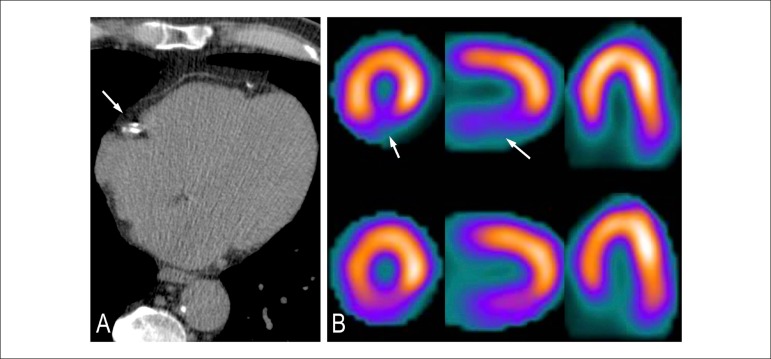
Patient with calcium score 11-100. (A) Discreet quantity of calcified plaques in the territory of the right coronary. Definite coronary artery disease, though discreet. (B) Myocardial scintigraphy shows ischemia in the inferior wall in region with attenuation by soft tissue.

High calcium score is an indicator of increased risk for cardiovascular events such as heart attack and cardiac death, with higher accuracy, alone or in joint assessments, than clinical risk scores (Figure 2 and 3). Its presence indicates a poor prognosis in these patients, reclassifying them to high-risk groups, regardless of population characteristics.^36-40^

The presence of a severely high calcium score is also related to a higher frequency of significant lesions, even in patients with normal scintigraphy.^61^ In persistently symptomatic patients with no perfusion alterations, extensive coronary calcification is correlated to the presence of significant lesions and may indicate the need for coronary angiography and percutaneous or surgical intervention.^62^

The presence of extensive coronary calcification is also associated to a higher incidence of significant obstructive disease and revascularization, even when the result of the provocative test is normal.^63-65^ Similarly, patients with altered scintigraphy have higher calcium score values compared to patients with normal perfusion exams.^41-43^

**Figure 4:**
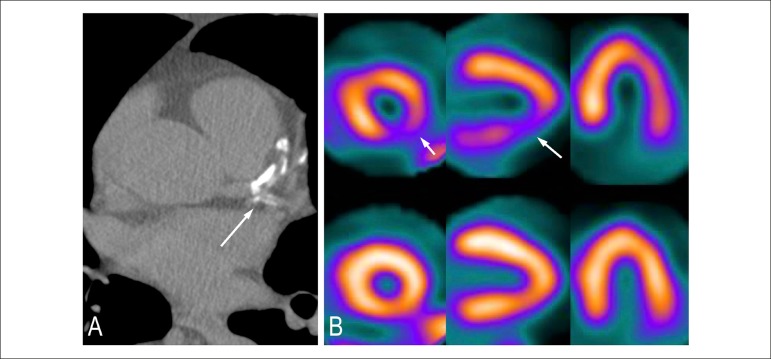
Patient with calcium score 101-400. (A) Moderate quantity of calcified plaque in the territories of the anterior descending and circumflex arteries. Moderate coronary arterial disease. (B) Myocardial scintigraphy with the presence of inferolateral ischemia.

**Figure 5:**
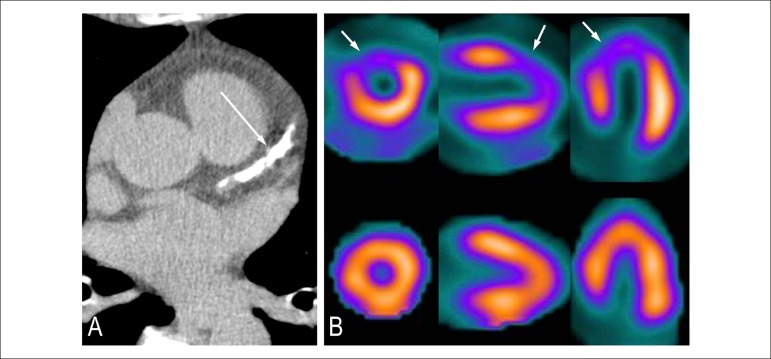
Patient with calcium score above 400. (A) Large quantity of calcified plaques in the territory of the anterior descending artery. Significant coronary artery disease. (B) Myocardial scintigraphy with presence of anterior and anteroseptal ischemia.

Increased coronary calcification is therefore able to correlate to the presence of obstructive lesion, even when the provocative test is normal, minimizing false-negative results with the combined use of both methods.^66^ This joint strategy is also able to refer patients who would benefit from additional investigation or invasive approaches (Figure 4 and 5).^69^

### Independent complementary information

Intermediate calcium score values are also important in the evaluation of coronary artery disease. CS proved complementary to MPS, regardless of the presence or absence of ischemia.^70^ In patients with normal scintigraphy, the presence of an altered score indicates higher risk, despite the good prognosis of normal scintigraphy; in turn, perfusion defect patients show greater risk of events and worse prognosis.^71^

The association of methods has greater diagnostic accuracy in detecting and warding off coronary disease and it more peremptorily determines the prognosis of these patients. Moreover, the presence of tests results, in parallel, allows better interpretation of the results by minimizing mistakes and improving method performance.^72,73^

In patients with normal scintigraphy, calcium score indicates subclinical disease and may assist in the exclusion of CAD or infer the presence of significant obstructive lesions.^74^ In persistently symptomatic patients, high calcium score was a significant coronary stenosis predictor despite scintigraphy results.^64^

The combined use of calcium score and scintigraphy becomes even more interesting if we take into account the prognostic value of methods for CAD. The occurrence of mortality and cardiac events is related to the severity of coronary calcification, regardless of scintigraphy results.^74-76^

However, the scintigraphy-established prognosis is characteristically short to medium term, in which a normal result is a predictor of good prognosis in this period, even in groups with elevated CS.^75,76^ On the other hand, calcium score can estimate the risk of longer-term periods -10 to 15 years - as noted recently, in which case, elevated coronary calcification overlaps the absence of perfusion defects.^39,40,74,76^

Moreover, an important aspect of the correlation between calcium score and MPS is the impact that the outcome of one method has on the interpretation of the other. Additional information and increased pre-test probability provided by coronary calcification exert positive influence on the interpretation of scintigraphy, improving accuracy and reducing the amount of equivocal results on the joint analysis of these two methods.^77^

### Exposure to radiation

With regards to radiation, calcium scoring method has a clear advantage by exposing the patient to lower doses than scintigraphy. Multicenter studies have shown that the average scintigraphy dose of radiation was higher than 10 millisieverts (mSv), with even higher averages in regions such as Latin America and Asia (15 mSv).^78,79^

Importantly, recent technological advances in scintigraphy, both in detectors and in image reconstruction software, enable these tests to be carried out with a much lower exposure to radiation compared to traditional techniques. The use of these advances allows examinations with an effective dose below 5 mSv with the use of ultra-low dose radiotracer protocols.^26,27,80,81^

On the other hand, calcium score has a low radiation dose, and in patients referred for scintigraphy, such dose is not too high as to impede the examination strategy. In studies evaluating the effective dose of the calcium score, the average was 2.5 mSv.^82,83^ Thus, calcium score is in a safe range for cancer risk.^84^

## Conclusion

Literature review shows that both calcium score and scintigraphy play an important role in the diagnostic evaluation of atherosclerotic heart disease. The possibility of removing extensive coronary disease by means of a calcium score zero, or indicating the presence of an extensive disease when it is severely increased, justifies the use of this method in the initial or joint evaluation, in asymptomatic patients with suspected CAD and in cardiovascular risk stratification. The evaluation of symptomatic low-risk patients, despite suggestive evidence, should be re-evaluated in upcoming guidelines.

Confirmation of the disease with the application of more specific methods and positive predictive value as myocardial perfusion scintigraphy is still fundamental in certain patients. Thus, although literature suggests that sequential or joint use of both methods is advantageous, more data are needed to establish a cost-effective strategy for diagnostic evaluation. It seems justifiable, therefore, from the standpoint of quality and accuracy of assessment and economic context of public health, that new studies continue researching the role of these important tools.
